# Diet quality, diet motives and nutrition literacy of vegans, vegetarians and semi-vegetarians

**DOI:** 10.1017/S1368980024001241

**Published:** 2024-06-03

**Authors:** Sapna Peruvemba, John Gieng, Susan Chen, Giselle Adriana Pereira Pignotti

**Affiliations:** Department of Nutrition, Food Science, and Packaging, San Jose State University, One Washington Square, San Jose, CA 95192-0058, USA

**Keywords:** Plant-based diet, Dietary intake, Health literacy, Motivation

## Abstract

**Objective:**

Limited research is available on how motivations to adopt plant-based diets and nutrition literacy influence diet quality. This study assessed diet quality, diet motives and nutrition literacy in vegans, vegetarians and semi-vegetarians and investigated predictors of dietary quality.

**Design:**

Cross-sectional study, participants completed an online survey about diet-related motives and nutrition literacy. Dietary intake was assessed with the Diet History Questionnaire III, and diet quality was calculated with the Healthy Eating Index (HEI)-2015. A one-way ANCOVA was used to compare diet quality, nutrition literacy and diet motives among diets. Hierarchical regression analysis was performed to identify significant predictors of diet quality.

**Setting:**

Online survey, participants were recruited through paid targeted social media (Facebook/Instagram) advertising.

**Participants:**

Adults following a plant-based diet, including 117 (52·5 %) vegans, 51 (22·9 %) vegetarians and 55 (24·6 %) semi-vegetarians.

**Results:**

Vegans had higher HEI-2015 scores (80·8 (sd 6·5), *P* < 0·001) compared to vegetarians (75·1 (sd 9·1)) and semi-vegetarians (76·8 (sd 7·5)). Most participants (74 %) had good nutrition literacy scores. Total nutrition literacy did not differ between groups, but vegans had higher vegetarian nutrition literacy than vegetarians and semi-vegetarians (*P* < 0·001). Ecological welfare, health and sensory appeal were highly important to all participants. Motives accounted for 12·8 % of the variance in diet quality scores. HEI-2015 scores were positively associated with motives of health and natural content, but negatively associated with weight control motivation (all *P* < 0·05).

**Conclusions:**

Individuals following plant-based dietary patterns have high diet quality and nutrition literacy. Messages valuing intrinsic over extrinsic factors may facilitate healthier dietary adherence in this population.

Plant-based diets are increasing in popularity, both in the public and in the scientific community^(1)^. While a plant-based diet is primarily focused on fruits and vegetables, grains, pulses, nuts and seeds, it may also include the reduction or exclusion of animal-based foods. For example, a vegan diet excludes all animal-based foods from the diet, a vegetarian diet excludes animal-based foods except for dairy and/or eggs, and a semi-vegetarian diet limits meat and flesh products^(2)^. According to the National Health and Nutrition Examination Survey dietary data from 2005 to 2018, 2·6 % of participants over 20 years of age consumed a vegetarian diet defined as a dietary pattern restricted of meat, poultry and seafood^(3)^.

Evaluating different types of dietary patterns provides an insight into health outcomes and chronic disease prevention efforts. Multiple approaches can be used to assess diet adequacy, such as evaluating the intake of nutrients, foods, dietary patterns or a combination. One common approach is the use of a diet quality index to measure an individual’s compliance with dietary guidelines to determine if an eating pattern is nutritionally adequate^(4)^. Studies comparing individuals following a plant-based diet (vegan, vegetarian and semi-vegetarian) and non-vegetarian diet found vegans had the highest diet quality and non-vegetarians had the lowest diet quality^(2,5)^. For instance, Clarys et al. (2014) found the highest diet quality scores in vegans (65·4 out of 100), followed by semi-vegetarians (59·4), pescatarians and lacto-ovo-vegetarians (58·7) and omnivores (54·2)^(2)^. This may be related to higher fruit, vegetable, whole grain and plant protein intake and lower sweet and soft drink intake observed among vegans compared with non-vegetarian diets^(6,7)^. In a meta-analysis of prospective cohort studies, diets that scored highly on multiple diet quality indices were associated with a decreased risk of CVD, cancer, type 2 diabetes, neurodegenerative disease and all-cause mortality^(8)^.

Understanding the motives behind food choices can inform public health strategies aimed at improving diet quality. Health, sensory appeal, convenience and price are shown to be the most important diet motives, or reasons for making food choices, in emerging adults from the US^(9)^. Ethics, such as animal welfare, environmental protection, religion and health, are also common motives for following a plant-based diet^(10–14)^. Vizcaino et al.^(14)^ found that those following a plant-based dietary pattern applied their beliefs through the foods they chose to consume. These beliefs included disapproval of animal agriculture and its resulting animal suffering, natural resource depletion and greenhouse gas emissions^(12)^. Health is a prominent motivational factor among those following plant-based and non-plant-based dietary patterns and may be considered for weight control, chronic disease prevention and management and naturality^(10,14)^.

A healthy diet is more likely to be adopted and maintained by individuals with nutrition-related skills, motives and knowledge^(15)^. Although almost a third of Americans have deficits in health literacy, nutrition literacy levels in the US have not been well established^(16)^. Nutrition literacy is the level of ability to evaluate and understand nutrition information and make educated dietary choices^(15)^. Hoffman^(17)^ hypothesises that vegetarians must develop greater nutrition literacy to justify their diet in a mostly non-vegetarian society and maintain nutritional adequacy. Although Leonard et al.^(18)^ found that vegetarians had higher nutrition knowledge scores than non-vegetarians, DeMay et al.^(19)^ and Saintila et al.^(20)^ reported no significant differences in nutrition knowledge between vegetarians and non-vegetarians.

Although many studies have characterised the diet quality of individuals following plant-based dietary patterns, there is a lack of evidence regarding the diet motives and nutrition literacy among different categories of plant-based dietary patterns. Additionally, previous research on the relationship between diet motives and diet quality has produced inconsistent results^(21–25)^. The health motive seems to be associated with beneficial dietary behaviours in individuals following plant-based dietary patterns^(11,13,21)^. Some studies discovered that being motivated by ethics was linked to higher diet quality, while being motivated by weight control and mood was linked to lower diet quality, but these results have not been replicated^(22,23)^. Few studies have focused on diet motives and its relationship to diet quality in individuals following a plant-based diet^(24,25)^. One study found that health-motivated vegetarians had higher HEI-2015 scores than those with other primary motivations for following a vegetarian diet (religion, family and environment)^(24)^. Whereas a study of physically active adults following a vegetarian diet reported that aspiration to improve performance was the only motive associated with better diet quality^(25)^. The primary objective of this study was to assess diet quality, diet motives and nutrition literacy across vegans, vegetarians and semi-vegetarians. The secondary objective was to investigate the predictors of dietary quality within this population. We hypothesised that being strongly motivated by health and having a high nutrition literacy would be associated with higher diet quality.

## Methods

In this cross-sectional study, there were three inclusion criteria (1) being 18 years or older, (2) residing within the US for at least one year and (3) self-identifying as vegan (defined as excluding all animal products), vegetarian (defined as excluding all animal products besides eggs and/or dairy) or semi-vegetarian (defined as excluding no animal products but limiting meat to ≤ 1× per week). Recruitment took place between August and October of 2021 using purposive sampling methods through paid Facebook and Instagram social media advertising. To target the paid advertisement to our specific audience, filters were used based on age range (18+), residential location (US) and user interests such as ‘Semi-Vegetarianism’, ‘Vegetarianism’ and ‘Plant-based diet’. As an incentive, participants were able to enter a raffle drawing for one of ten $20 gift cards. Participants who completed the FFQ had the option to download a detailed analysis of their diet.

Participants completed an online questionnaire on the Qualtrics survey platform, featuring demographic questions, the revised Food Choice Questionnaire (FCQ),^(26)^ the Nutrition Literacy Assessment Instrument (NLit)^(15)^ and questions related to vegetarian nutrition knowledge. The median time to complete the survey was 21·6 min (interquartile range: 16·9–26·9). The demographic questions included sex at birth, age, race/ethnicity, educational level and household income. Participants also responded to questions regarding foods excluded from their diet (meat, chicken, fish and seafood, eggs and dairy products), which was used to categorise participants into vegan, vegetarian or semi-vegetarian groups and duration of dietary adherence.

Food choice motives were assessed using the revised FCQ, which Lindeman and Väänänen^(26)^ adapted to include three new ethical scales. The FCQ is used to systematically assess motives that influence dietary choices. This version included 44 items and 11 subscales: health (6 items), mood (6 items), convenience (5 items), sensory appeal (4 items), natural content (3 items; e.g. avoidance of additives, artificial ingredients), price (3 items), weight control (3 items), familiarity (3 items), ecological welfare (5 items), political values (4 items) and religion (2 items)^(26)^. Participants ranked each food choice statement on a five-point Likert scale (e.g. ‘It is important to me that the food I eat on a typical day keeps me healthy’, where 1 = Strongly disagree, 2 = Somewhat disagree, 3 = Neither agree nor disagree, 4 = Somewhat agree and 5 = Strongly agree). For each scale, an average of the values for each of the statements was used to create a score ranging from 1 to 5 for each participant.

Nutrition literacy was assessed with the NLit, which has been validated to measure dietary knowledge and nutrition-related skills among adult populations^(15)^. The NLit included 64 items and six domains: nutrition & health (10 items), energy sources in food (10 items), household food measurement (9 items), food label & numeracy (10 items), food groups (16 items) and consumer skills (9 items). Data for each item were coded as correct/incorrect, with missing answers coded as incorrect. Scores ranged from 0 to 64, with scores of 44 or below indicating the likelihood of poor nutrition literacy, scores between 45 and 57 indicating the possibility of poor nutrition literacy and scores of 58 and above indicating the likelihood of good nutrition literacy^(15)^. Vegetarian nutrition literacy questions were developed to address nutrition knowledge specific to plant-based dietary patterns. The vegetarian nutrition literacy question topics included B_12_ food sources (1 item), plant-based calcium (1 item), plant protein (1 item), fortified foods (1 item) and non-dairy milk choices (1 item). Data for each item were coded as correct/incorrect, with missing answers coded as incorrect. Scores ranged from 0 to 5.

At the end of the survey, participants provided their email addresses to receive a link to the Diet History Questionnaire III, available on the National Cancer Institute website, with a unique login and password^(27)^. The Diet History Questionnaire III is a validated FFQ used to assess food and supplement intake. It includes 135 questions regarding food and beverages and 26 questions regarding dietary supplements^(27)^. Participants chose their consumption frequency from several categories (1 time in the past month, 2–3 times in the past month, 1 time per week, 2 times per week, 3–4 times per week, 5–6 times per week, 1 time per day, 2 or more times per day). The Diet History Questionnaire III asked participants to report their age and gender at the start of the questionnaire to assign predetermined portion sizes and provide a mean nutrient or food group value for each food on the DHQ^(27)^. These values were used to determine the Healthy Eating Index (HEI) 2015 score for each participant ranging from 0 to 100. The HEI score is a measurement of diet quality that determines adherence to the *US Dietary Guidelines for Americans* ^(28)^.

Using Pearson’s chi-square tests, we examined whether age, gender, race/ethnicity, education, income and duration of the diet differed among the three types of diet: vegans, vegetarians and semi-vegetarians. A one-way ANOVA with a Dunn–Bonferroni post hoc test was conducted to compare diet quality, nutrition literacy, vegetarian nutrition literacy and food choice motive scores among diet groups. We assessed the predictors of diet quality using a three-step hierarchical regression analysis among the whole sample of vegans, vegetarians and semi-vegetarians. Step 1 included sociodemographic characteristics (age, sex, education, income and race/ethnicity), step 2 included nutrition literacy and vegetarian nutrition literacy, and step 3 included the 11 motives from the FCQ. All statistical analyses were performed using SPSS version 27. *A priori* power analysis for linear regression was performed using G. Power version 3.1. A minimum of 157 participants were required to test the association between 20 predictors and diet quality score considering a 0·15 effect size, 80 % power and 0·05 alpha^(29)^.

## Results

A total of 972 respondents consented to participate, of which 921 met the inclusion criteria and 387 provided their email addresses to participate in the dietary intake assessment. The final sample consisted of 223 participants who completed all portions of this study and were included in the data analyses. Participants were categorised into three dietary pattern groups: vegan (52·5 %), vegetarian (22·9 %) and semi-vegetarian (24·6 %). As shown in Table 1, the majority of participants were female (87 %), aged 40 and above (72 %), white non-Hispanic (86 %), college-graduates (78 %) and had a household income at or above $50 000 (67 %). When comparing the three diet groups, no significant differences were observed in any demographic characteristics. The vegetarian group was more likely to have followed their diet for 10 years or longer (65 %) than the vegan group (36 %) and semi-vegetarian group (39 %; *P* = 0·010).

**Table 1:** Demographic characteristics of vegans, vegetarians, and semi-vegetarians

	Total (*n* 223)		Vegan (*n* 117)		Vegetarians (*n* 51)		Semi-vegetarians (*n* 55)		*P*-value^*^
	*n*	%	*n*	%	*n*	%	*n*	%	
Age									0·422
18–39 years	63	28·3	27	23·1	17	33·3	19	334·5	
40–59 years	75	33·6	41	35	18	35·3	16	29·1	
60 or more years	85	38·1	49	41·9	16	31·4	20	36·4	
Sex									0·168
Male	29	13	15	12·8	10	19·6	4	7·3	
Female	194	87	102	87·2	41	80·4	51	92·7	
Race/Ethnicity									0·131
White, non-Hispanic	188	85·8	103	90·4	41	82	44	80	
Asian/Black/Hispanic/Multi-race/Other	31	14·2	11	9·6	9	18	11	20	
Education									0·794
No college degree	49	22	28	23·9	9	17·6	12	21·8	
College degree	68	30·5	36	30·8	15	29·4	17	30·9	
Graduate degree	106	47·5	53	45·3	27	52·9	26	47·3	
Household income									0·842
Less than $50 000	72	32·9	36	31·3	17	34	19	35·2	
$50 000–$99 999	78	35·6	39	33·9	18	36	21	38·9	
$100 000 or more	69	31·5	40	34·8	15	30	14	25·9	
Duration of diet									**0·010**
2 years or less	37	16·7	20	17·1	3	5·9	14	25·9	
3–5 years	46	20·7	29	24·8	7	13·7	10	18·5	
6–10 years	43	19·4	26	22·2	8	15·7	9	16·7	
More than 10 years	96	43·2	42	35·9	33	64·7	21	38·9	

*P* values lower than 0.05 were boldface to highlight statistically significant results.

* Chi-square.

Table 2 shows differences in diet quality, nutrition literacy and food choice motives by diet groups. On average, participants had high scores for diet quality (78·5 (sd 7·8) out of 100), nutrition literacy (58·9 (sd 3·3) out of 64) and vegetarian nutrition literacy (5·5 (sd 0·7) out of 6). Vegans had a higher total HEI-2015 score than vegetarians and semi-vegetarians (*P* < 0·001). This was mostly attributed to higher scores in total fruits, total vegetables, fatty acids and saturated fat. Regarding nutrition literacy, 74 % of our sample had good nutrition literacy (≥ 58), 25·6 % had possibly poor nutrition literacy (45–57), and 0·4 % had poor nutrition literacy (≤ 44). There were no significant differences in nutrition literacy among diet groups. However, the vegan group had a higher vegetarian nutrition literacy score compared to the vegetarian and semi-vegetarian group (*P* < 0·001). Overall, ecological welfare, health and sensory appeal were the most important motives to participants. Specifically, vegans valued ecological welfare significantly more than did semi-vegetarians (4·43 (sd 0·46) *v*. 4·22 (sd 0·6), *P* = 0·048), while semi-vegetarians valued weight control (3·68 (sd 0·83) *v*. 3·24 (sd 0·78), *P* = 0·028) and health (4·28 (sd 0·52) *v*. 4·01 (sd 0·51), *P* = 0·028) significantly more than did vegetarians. Moreover, semi-vegetarians valued familiarity significantly more than did vegans (3·03 (sd 0·95) *v*. 2·59 (sd 0·87), *P* = 0·018).

**Table 2:** Diet quality, nutrition literacy, and food choice motives by diet type

	Total (*n* 223)		Vegan (*n* 117)		Vegetarian (*n* 51)		Semi-vegetarian (*n* 55)		*P*-value^*^
	Mean	sd	Mean	sd	Mean	sd	Mean	sd	
Total HEI-2015 (0–100)	78·5	7·8	80·8	6·5	75·3	9·0^†^	76·8	7·5^†^	**< 0·001**
Total vegetables (0–5)	4·8	0·5	4·9	0·3	4·8	0·6	4·6	0·8^†^	**0·001**
Greens and beans (0–5)	4·9	0·5	5·0	0·1	4·9	0·6	4·8	0·7^†^	**0·008**
Total fruits (0–5)	4·4	1·1	4·6	0·9	3·9	1·4^†^	4·2	1·1	**0·001**
Whole fruits (0–5)	4·8	0·8	4·9	0·6	4·5	1·1^†^	4·8	0·7	**0·020**
Whole grains (0–10)	6·1	2·8	6·7	2·8	5·6	2·9	5·3	2·6^†^	**0·005**
Dairy (0–10)	4·3	2·7	3·5	2·5	5·1	2·5^†^	5·5	2·7^†^	**< 0·001**
Total protein (0–5)	4·6	0·8	4·7	0·7	4·4	0·9	4·6	0·8	0·179
Seafood and plant protein (0–5)	5·0	0·3	5·0	0·3	4·9	0·3	5·0	0·1	0·626
Fatty acids (0–10)	8·6	2·4	9·6	1·4	7·6	2·9^†^	7·6	2·7^†^	**< 0·001**
Sodium (0–10)	4·5	2·8	4·3	2·8	4·8	2·6	4·7	3·0	0·529
Refined grains (0–10)	8·7	2·3	8·9	2·1	7·7	3·1^†^	9·0	1·7^‡^	**0·006**
Saturated fats (0–10)	8·8	2·1	9·8	0·6	7·7	2·8^†^	7·9	2·5^†^	**< 0·001**
Added sugars (0–10)	9·0	1·6	9·1	1·7	9·3	0·8	8·7	1·9	0·103
Nutrition literacy (0–64)	58·9	3·3	59·2	3·1	58·8	3·6	58·4	3·5	0·381
Vegetarian nutrition literacy (0–5)	4·6	0·6	4·8	0·6	4·4	0·9^†^	4·2	0·8^†^	**< 0·001**
FCQ (1–5)									
Ecological welfare	4·36	0·52	4·43	0·46	4·34	0·52	4·22	0·6^†^	**0·048**
Health	4·12	0·54	4·09	0·55	4·01	0·51	4·28	0·52^‡^	**0·028**
Sensory appeal	4·12	0·52	4·12	0·5	4·03	0·6	4·23	0·47	0·125
Natural content	4·05	0·91	4·05	0·98	3·86	0·78	4·22	0·84	0·137
Convenience	3·52	0·74	3·55	0·74	3·33	0·69	3·62	0·75	0·094
Weight control	3·47	0·87	3·48	0·90	3·24	0·78	3·68	0·83^‡^	**0·028**
Political values	3·41	0·74	3·38	0·72	3·33	0·74	3·53	0·79	0·359
Mood	3·38	0·68	3·33	0·64	3·29	0·73	3·58	0·70	**0·046** ^§^
Price	3·35	0·84	3·29	0·81	3·40	0·84	3·45	0·92	0·465
Familiarity	2·72	0·93	2·59	0·87	2·69	0·97	3·03	0·95^†^	**0·018**
Religion	2·61	1·13	2·58	1·06	2·50	1·25	2·78	1·16	0·406

HEI, Healthy Eating Index-2015; FCQ, Food Choice Questionnaire (1 – strongly disagree, 5 – strongly agree).

*P* values lower than 0.05 were boldface to highlight statistically significant results.

* Mean values between diet types were compared using ANOVA.

† Significantly different from vegans as determined by the Dunn–Bonferroni post hoc method.

‡ Significantly different from vegetarians as determined by the Dunn–Bonferroni post hoc method.

§ NS in the post hoc analysis.

Table 3 shows the results of the three-step hierarchical regression analysis of the effects of food choice motives on overall diet quality (total HEI-2015 score). The analysis controlled for sociodemographic variables in step 1 and further adjusted for nutrition and vegetarian nutrition literacy in step 2. The final model that included all variables explained 26·5 % of the variance in diet quality (*P* < 0·001), while motives alone accounted for 12·8 % of the variance, and demographics accounted for 6·8 %. Notably, nutrition and vegetarian nutrition literacy did not significantly predict diet quality. In the fully adjusted model, increased importance of health was associated with a 3·4-point increase in HEI-2015 score (*P* = 0·004) and higher importance of natural content motivation was associated with a 1·7-point increase in HEI-2015 score (*P* = 0·014). In contrast, greater importance of weight control motivation was associated with lower HEI-2015 scores (*P* = 0·025).

**Table 3:** Predictors of diet quality among the overall sample of vegans, vegetarians, and semi-vegetarians (*n* 223)

Variable ^a,b^	Model 1		Model 2		Model 3	
	Unstandardised *β*	*P*-value	Unstandardised *β*	*P*-value	Unstandardised *β*	*P*-value
Constant	72·12	**< 0·001**	77·54	**< 0·001**	63·39	**< 0·001**
Age	0·04	0·180	0·04	0·171	0·02	0·601
Sex (female)	−2·02	0·199	−1·81	0·253	−1·35	0·363
Education	0·44	0·295	0·49	0·247	0·35	0·373
Income	0·65	**0·049**	0·70	**0·036**	0·62	0·058
Race/ethnicity (all other)	−2·27	0·138	−2·36	0·145	−1·80	0·259
Nutrition Literacy			−0·22	0·203	−0·17	0·313
Vegetarian Nutrition Literacy			1·51	0·080	1·29	0·124
Convenience					0·15	0·850
Health					3·44	**0·004**
Natural content					1·71	**0·014**
Sensory appeal					−2·33	0·054
Mood					1·01	0·329
Familiarity					−1·18	0·070
Weight control					−1·54	**0·025**
Price					−0·59	0·403
Ecological welfare					2·08	0·071
Political values					−0·65	0·425
Religion					0·83	0·083
Model Total R^2^	0·068	**0·012**	0·086	**0·009**	0·265	**< 0·001**
Model ΔR^2^			0·019	0·128	0·179	**< 0·001**

*P* values lower than 0.05 were boldface to highlight statistically significant results.

a Three-step hierarchical regression analysis with Total Healthy Eating Index-2015 as the dependent variable.

b Reference groups for categorical variables – Sex: male; Race/ethnicity: non-Hispanic White.

## Discussion

The primary aim of this study was to assess and compare the diet quality, motives and nutrition literacy of vegans, vegetarians and semi-vegetarians. Overall, participants had a high diet quality. When comparing plant-based diet groups, vegans had the highest diet quality, while nutrition literacy levels were similar across groups. Nonetheless, vegans had a higher vegetarian nutrition literacy score compared to vegetarians and semi-vegetarians. In addition, the most valued motives in the sample were ecological welfare, health and sensory appeal. The secondary aim of the study was to identify the predictors of diet quality. Our findings suggest that intrinsic motivations to follow a plant-based diet are associated with greater diet quality, while extrinsic motivations are associated with poorer diet quality. An intrinsically motivated person performs a behaviour because it is personally rewarding to them, for example the food choice aligns with their beliefs and values, whereas an extrinsically motivated person performs a behaviour to gain an external reward, such as choosing food for lower body weight, improved mood or cheaper prices^(30)^. In our study, individuals following plant-based dietary patterns who were motivated by health or natural content had higher diet quality. On the other hand, those who were motivated by weight control had lower diet quality.

When comparing vegan, vegetarian and semi-vegetarian diets, vegans were found to have the highest diet quality scores, which is consistent with other studies^(2,5,7,24)^. Mean HEI scores in the literature ranged from 65·4 to 70·9 in vegans, 58·7 to 60·9 in vegetarians and 59·4 in semi-vegetarians^(2,24)^. In comparison, the mean HEI-2015 score of the US population was 56·6^(28)^. Similar to our findings, previous research found that vegans consumed more fruits, vegetables, whole grains and legumes, and fewer products high in saturated fat^(2,6,7)^.

Nutrition literacy was favourable in this population, with no differences in total nutrition literacy among groups, but higher vegetarian nutrition literacy in vegans. Studies characterising nutrition literacy in individuals following a plant-based diet are limited, and existing findings are contradictory. Saintila et al.^(20)^ reported no significant differences in the level of nutritional knowledge between vegetarian and non-vegetarian diets. However, Leonard et al.^(18)^ observed that vegetarians and semi-vegetarians had higher total knowledge scores than non-vegetarians. Similarly, Hoffman et al.^(17)^ discovered that the more restrictive the diet, the greater the nutrition knowledge, with vegans scoring highest, followed by ovo-lacto-vegetarians. DeMay et al.^(19)^ found that vegetarians (including vegans) scored higher on questions relevant to their diet compared to non-vegetarians, which may support the greater vegetarian nutrition literacy seen in vegans in the present study. Those following a vegan or vegetarian diet may develop greater nutrition knowledge to justify their diet in a mostly non-vegetarian society and to maintain a nutritionally adequate diet^(17)^.

When making dietary choices, vegans valued ecological welfare more than semi-vegetarians did. This agrees with previous findings, which indicate that vegans and vegetarians view their dietary patterns as a manifestation of their animal and environmental values^(10,21,31)^. Many vegans and vegetarians endorsed disgust of animal products or affinity for vegetarian alternatives and therefore valued taste preferences more than low-meat consumers^(31)^ and semi-vegetarians^(32)^. However, our study found no differences in sensory appeal as a food choice motivation among vegans, vegetarians and semi-vegetarians, whereas semi-vegetarians valued weight control and health more than vegetarians, a finding that is consistent with the literature^(21,32)^.

Contradicting our study’s hypothesis, nutrition literacy and vegetarian nutrition literacy were not associated with diet quality. While no other study has evaluated this relationship among adults following plant-based dietary patterns, a systematic review of 29 relevant studies found that most showed a significant positive, but weak correlation between nutrition knowledge and dietary intake^(33)^. However, at the time, few studies have assessed this relationship using a validated and comprehensive nutrition literacy instrument. Nutrition literacy assessed using the NLit tool has been shown to predict diet quality among adults with chronic disease^(15,34)^. Taylor et al.^(34)^ observed that a high nutrition literacy was associated with healthful dietary practices including lower sugar intake, improved energy balance and increased nut and vegetable intake. Considering our participants were highly educated, at or above middle class, with high nutrition literacy, the low variability in NLit scores may explain the lack of association between diet quality and nutrition literacy.

The link between diet motivation and diet quality can be observed in those following plant-based dietary patterns. Increased importance of health and natural content motives was positively associated with HEI-2015 scores. Generally, the literature shows that health-motivated individuals are likely to have higher diet quality, and this was seen in individuals following plant-based dietary patterns as well^(22,24,35)^. However, studies assessing plant-based diets did not utilise the FCQ^(22,24)^ and therefore did not measure natural content, related to the presence of additives and artificial ingredients, as a separate motive from health. Our results also show that greater importance of weight control was associated with decreased HEI-2015 scores. Inconsistent with the present findings, a recent study found that increased importance of weight control was correlated with increased diet quality^(23)^. However, among those following plant-based dietary patterns, stronger weight-loss motivation was related to higher meat intake^(21)^. While weight control is related to health, it is driven by the external reward of a goal weight, which is subject to change, thereby altering dietary behaviours and quality.

To date, few studies have investigated the overall diet quality, diet motives and nutrition literacy in individuals following plant-based dietary patterns. While Torna et al.^(24)^ have investigated the relationship between diet motives and diet quality in vegetarians, our study is novel in that it differentiated between vegans and vegetarians and included semi-vegetarians. Those following plant-based dietary patterns often report multiple motives to dietary adherence and using the FCQ allowed several motives to be measured rather than requiring participants to choose their top motive^(26,31)^. Our sample is reflective of vegetarians in Western societies, who tend to be mostly (> 75 %) female, non-Hispanic white, middle-aged (40–60 years), with more than a high school education and a household income within the US middle-class range^(2,31,36)^. To our knowledge, this is the first study to measure nutrition literacy in individuals following plant-based dietary patterns using the validated NLit instrument. However, as the NLit has not been adapted for restrictive diets, it is unclear whether this instrument is appropriate to measure nutrition literacy in this population. Additionally, nutrition literacy levels in the US population have not been determined, as most studies to date were conducted in adults with chronic conditions,^(15,34)^ so interpretation of our nutrition literacy scores is limited. This study also did not include a control group of non-plant-based diets. The time-intensive design of this study, which included a 25-minute survey and an hour-long validated FFQ, may have limited our findings. From the overall sample, only 35 % of survey respondents completed the DHQ and were included in this study. Therefore, this study is prone to selection bias as more motivated health-conscious participants may have completed the study. However, the demographic variables of study participants who completed the DHQ were similar to those who did not. Nonetheless, results should be interpreted with caution due to limited generalisability.

The present study supports that individuals who were following plant-based dietary patterns had a high diet quality and nutrition literacy, with those following a vegan dietary pattern performing better than vegetarians and semi-vegetarians. In addition, this study underscores the importance of integrating diet motives and nutrition literacy in future research and public health settings alike to better understand and promote plant-based diets. Organisations can develop more influential public health campaigns that promote plant-based diets based on top-rated motives and existing nutrition knowledge. In patients adopting plant-based diets, healthcare professionals can encourage patients to focus on internal, personal motivations rather than external factors regarding diet. Shifting the clinical approach from focusing solely on weight control to including health and natural content as considerations may facilitate better dietary quality. Future research studies may benefit from including a larger group of participants and separating plant-based dietary patterns into different groups (vegans, vegetarians and semi-vegetarians) to account for differences in characteristics as shown in this study. There is no consensus regarding the definition of the semi-vegetarian diet and its differences from flexitarian and low-meat diets. Defining these terms more clearly will allow researchers to better establish diet quality, nutrition literacy and motivations in individuals following these diets.
